# Disparities in Pancreatic Ductal Adenocarcinoma—The Significance of Hispanic Ethnicity, Subgroup Analysis, and Treatment Facility on Clinical Outcomes

**DOI:** 10.1002/cam4.3042

**Published:** 2020-04-13

**Authors:** Andrea N. Riner, Patrick W. Underwood, Kai Yang, Kelly M. Herremans, Miles E. Cameron, Srikar Chamala, Peihua Qiu, Thomas J. George, Jennifer B. Permuth, Nipun B. Merchant, Jose G. Trevino

**Affiliations:** ^1^ Department of Surgery University of Florida College of Medicine Gainesville FL USA; ^2^ Department of Biostatistics University of Florida College of Medicine Gainesville FL USA; ^3^ Department of Physical Therapy University of Florida College of Medicine Gainesville FL USA; ^4^ Department of Pathology, Immunology and Laboratory Medicine University of Florida College of Medicine Gainesville FL USA; ^5^ Division of Hematology and Oncology Department of Medicine University of Florida College of Medicine Gainesville FL USA; ^6^ Departments of Cancer Epidemiology and Gastrointestinal Oncology Moffitt Cancer Center and Research Institute Tampa FL USA; ^7^ Department of Surgery University of Miami College of Medicine Miami FL USA

**Keywords:** pancreatic ductal adenocarcinoma, survival

## Abstract

**Background:**

Disparities exist among patients with pancreatic ductal adenocarcinoma (PDAC). Non‐White race is regarded as a negative predictor of expected treatment and overall survival. Data suggest that Academic Research Programs (ARP) provide better outcomes for minorities, but ethnic/minority outcomes are underreported. We hypothesize that outcomes among racially/ethnically diverse PDAC patients may be influenced by treatment facility.

**Methods:**

The National Cancer Database was used to identify 170,327 patients diagnosed with PDAC between 2004 and 2015. Cox proportional‐hazard regression was used to compare survival between race/ethnic groups across facilities.

**Results:**

In unadjusted models, compared to non‐Hispanic Whites (NHW), non‐Hispanic Blacks (NHB) had the worst overall survival (HR = 1.05, 95%CI: 1.03‐1.06, *P* < .001) and Hispanics had the best overall survival (HR = 0.92, 95%CI: 0.90‐0.94, *P* < .001). After controlling for socioeconomic and clinical covariates, NHB (HR = 0.95, 95%CI: 0.93‐0.96, *P* < .001) had better overall survival compared to NHW, and Hispanics continued to have the best comparative outcomes (HR = 0.84, 95%CI: 0.82‐0.86, *P* < .001). Among Hispanics, Dominicans and South/Central Americans lived the longest, at 10.25 and 9.82 months, respectively. The improved survival in Hispanics was most pronounced at ARP (HR = 0.80, 95%CI: 0.77‐0.84, *P* < .001) and Integrated Network Cancer Programs (HR = 0.78, 95%CI: 0.73‐0.84, *P* < .001). NHB had improved survival over NHW at Comprehensive Community Care Programs (HR = 0.96, 95%CI: 0.93‐0.98, *P* = .002) and ARP (HR = 0.96, 95%CI: 0.94‐0.98, *P* = .001), which was influenced by income, education, and surgical resection.

**Conclusion:**

Survival was improved at ARP for all populations. Hispanics had the best comparative overall survival. NHB had improved overall survival at higher volume centers, but this was dependent upon income, education, and surgical resection.

## Graphical abstract

Overall survival with pancreatic ductal adenocarcinoma is influenced by where treatment is received, for all races/ethnicities. While racial/ethnic disparities affect overall survival, further subclassification of Hispanics into regions of origin has significant implications in survival and clinical outcomes with pancreatic ductal adenocarcinoma.

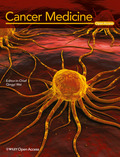

**Abbreviations**
ARP: Academic and Research Program
CCCP: Comprehensive Community Cancer Program
CCP: Community Cancer Program
INCP: Integrated Network Cancer Program
NCDB: National Cancer Database
NHB: non‐hispanic black
NHW: non‐hispanic white
OSSHO: other specified Spanish or Hispanic origin
PDAC: pancreatic ductal adenocarcinoma
SCA: South or Central American, except Brazil
SHLNOS: Spanish, Hispanic, and Latino not otherwise specified
SSO: Spanish surname only

## 1 INTRODUCTION

Pancreatic ductal adenocarcinoma (PDAC) is one of the most lethal malignancies. The 5‐year survival rate remains at a dismal 9%, and incidence and mortality rates are nearly equal.^1^, ^2^ Further complicating the disease and clinical care are a variety of racial and ethnic disparities that affect both incidence and mortality rates. Non‐Hispanic Blacks (NHB) have the highest incidence (15.9 per 100 000) and death rates (13.7 per 100 000), followed by non‐Hispanic Whites (NHW) (incidence rate of 12.6 per 100 000 and death rate of 11.1 per 100 000) and Hispanics (incidence rate 10.6 per 100 000 and death rate of 8.5 per 100 000).^3^, ^4^ The cause for disparities among different ethnic or racial groups is not fully understood, but is likely multifactorial.

Disparities are seen in treatment and resection rates, as well as survival for patients with PDAC.^5^, ^6^, ^7^ Non‐White race is an independent negative predictor of receiving expected treatment for clinical stage.^8^ Although resection is offered at similar rates across racial or ethnic groups, NHB are more likely to refuse surgery.^7^, ^9^ Patients are also more likely to refuse surgery if they receive care at a non‐Academic Research Program (ARP).^10^ High‐volume academic centers have higher expected treatment adherence for clinical stage, but disparities remain among racial or ethnic groups.^8^ Overall survival is also improved at ARP despite treating patients with more advanced or aggressive disease.^11^ Survival is clearly improved when patients receive appropriate treatment based on disease stage, including surgical resection for locoregional disease, but expected treatment adherence does not account for all racial or ethnic disparities observed.

Little data are published on disparities in overall survival in Hispanic patients with PDAC.^7^ While outcomes among Hispanics with PDAC may generally be improved compared to non‐Hispanics, a more in‐depth understanding of this phenomenon is warranted as Hispanics represent a diverse ethnic group with a variety of ancestral heritages, including ancestry from indigenous American, African, Asian, and European peoples. The clinical outcomes from patients with PDAC from these varied ancestries have not been defined. Identification of population subgroups with particularly indolent or aggressive pancreatic cancer may lead to further understanding of tumor biology and pathogenesis, and ultimately an improved ability to provide precision medicine. While better clinical outcomes are suggested at ARP for minority populations,^11^ again, the differentiation among Hispanics has not been adequately assessed. We sought to determine if outcomes among diverse racial or ethnic patients with PDAC are influenced by where patients receive their oncologic care, with a focus on Hispanic subgroups.

## 2 METHODS

### 2.1 Study population

A retrospective review of the National Cancer Database (NCDB) was performed to identify patients diagnosed with PDAC from 2004 to 2015. The NCDB is a hospital‐based registry and joint project of the Commission on Cancer (CoC) of the American College of Surgeons and the American Cancer Society that captures 70% of all diagnosed malignancies in the US annually.^12^ Access to the de‐identified NCDB Participant User Data File was requested via the CoC. Institutional Review Board approval was not required. We identified 197 350 patients with histologically proven PDAC. Additional data included treatment facility type and patient characteristics including socioeconomic factors, geography, clinical features, surgical resection, and survival. After removing cases with unknown survival status or multiple missing covariates, 170 327 patients were included in the final analysis.

### 2.2 Study variables

Patient characteristics included age, sex, race and ethnicity, median household income, level of education, insurance, facility location, distance from treatment facility, and population density in their area of residence. Age was analyzed as a continuous variable. Sex was defined as male or female. Race/ethnicity was categorized as non‐Hispanic White, non‐Hispanic Black, and Hispanic, which was further characterized by country or region of origin, including Dominican, South and Central American, Mexican, Puerto Rican, Cuban, other specified Hispanic/Spanish origin, Not Otherwise Specified, or Spanish surname only. Median household income was based on median income in the patient's residential zip code and level of education was based on percentage of adults who did not graduate from high school in the patient's zip code, according to the 2000 US Census. Insurance coverage was classified as uninsured, private, Medicaid, and Medicare. Facility location was based on geographic region within the US. Distance from treatment facility was analyzed as a continuous variable. Population density was categorized as metropolitan, metropolitan adjacent, non‐metropolitan adjacent, and rural. Tumor grade was not included as over 60% of cases had missing data.

Facility type was categorized as Community Cancer Program (CCP), Comprehensive Community Cancer Program (CCCP), Academic Research Program (ARP), and Integrated Network Cancer Program (INCP). CCP treat 100 to 500 newly diagnosed cancer patients annually, and may refer patients to another facility. Comprehensive Community Cancer Programs treat more than 500 newly diagnosed patients annually. Academic Research Program treat more than 500 newly diagnosed patients annually and offer postgraduate medical education. Academic Research Program include National Cancer Institute designated comprehensive cancer centers. Integrated Network Cancer Program include at least one CoC‐accredited cancer program under an umbrella program that must meet performance expectations for quality measures, participate in clinical research, with no required quota for newly diagnosed patients, and optional resident training. Many INCP are large hospital networks and hybrids of ARP with CCP.

Surgical resection was a dichotomous variable with “yes” including local tumor excision or pancreatectomy. Staging was defined between I and IV, based on the American Joint Committee on Cancer staging. Charlson‐Deyo comorbidity score was utilized as a measure of overall health status.^13^, ^14^

The outcome of interest was overall survival, defined by the number of months between the date of diagnosis and when the patient was last contacted or died.

### 2.3 Statistical analysis

Statistical analyses were conducted using *R‐3.4.3*.^15^ A *P* < 0.05 was considered significant. Descriptive statistics were calculated based on information from 170 327 PDAC patients. Relationships between survival time, defined by last contact or death in months from date of diagnosis, and the two covariates of interest (race/ethnicity and facility type) were investigated. Overall survival was evaluated using both Kaplan‐Meier estimator and univariate Cox proportional‐hazards models. The multivariate Cox proportional‐hazards model was fit to analyze the patients' survival time, and backward model selection was used to determine covariates for inclusion. The final Cox model was used to compare survival between race/ethnic groups across facility types while adjusting for patient sex, age, income, level of education, insurance, geographic variables, Charlson‐Deyo score, cancer stage, and surgical resection. Results are reported in hazard ratios (HRs) with 95% confidence intervals. We obtained median survival for each race/ethnic group, stratified by facility type. Pairwise comparisons were performed to determine the facility type and the race/ethnic group in which patients had the best survival. Unless otherwise stated, all statistical tests were two‐sided.

## 3 RESULTS

### 3.1 Patient population

A total of 170 327 patients were included in the final analysis (Table 1). ARP (14%) and INCP (16%) treated more NHB patients than CCP and CCCP (10% each), while all facilities treated similar percentage of Hispanics (4%‐6%). Most patients sought care at ARP (43.9%) and CCCP (37.6%), followed by INCP (11.4%) and CCP (7.0%). The age, sex distribution, and Charlson‐Deyo score were similar across the various facility types. ARP and INCP treated a higher percentage of NHB (14% and 16% of their overall patient population, respectively) compared to community programs (10% each). ARP, INCP, and CCCP treated more patients from metropolitan areas (83%, 87%, and 81%, respectively) compared to CCP (69%). CCP treated the lowest percentage of patients living in zip codes with the highest income bracket (24%), while ARP treated the highest percentage of patients in the highest income bracket (35%). CCP treated a higher percentage of patients from zip codes with lower educational attainment, while CCCP and ARP treated a higher percentage of patients from zip codes with more educational attainment. ARP provided care for a higher percentage of patients with private insurance (36%) and a lower percentage of patients with Medicare (55%). Patients treated at ARP traveled approximately twice as far to their treatment facilities (55.7 ± 162.3 miles), compared to other facility types. Community programs were more utilized in the Pacific region, whereas ARP was more utilized in the Middle Atlantic and INCP in the South Atlantic. ARP treated the highest percentage of patients diagnosed with stage II or III (potentially curable) disease (36%) and the lowest percentage of patients with stage IV (palliative) disease (41%), while CCP treated 19% with stage II disease and 64% with stage IV disease.

**TABLE 1:** Patient characteristics by facility type

Variable	All facilities (n = 170 327)	CCP (n = 11 988, 7.0%)	CCCP (n = 64 120, 37.6%)	ARP (n = 74 841, 43.9%)	INCP (n = 19 378, 11.4%)
	n (%) or mean ± SD	n (%) or mean ± SD	n (%) or mean ± SD	n (%) or mean ± SD	n (%) or mean ± SD
Race					
Non‐Hispanic White	140 871 (83%)	10 211 (85%)	54 748 (85%)	60 806 (81%)	15 106 (78%)
Non‐Hispanic Black	21 115 (12%)	1258 (10%)	6395 (10%)	10 395 (14%)	3067 (16%)
Hispanic	8341 (5%)	519 (4%)	2977 (5%)	3640 (5%)	1205 (6%)
Dominican	168 (2%)	11 (2%)	23 (1%)	119 (3%)	15 (1%)
SCA	667 (8%)	40 (8%)	178 (6%)	320 (9%)	129 (11%)
Cuban	502 (6%)	7 (1%)	120 (4%)	98 (3%)	277 (23%)
Puerto Rican	380 (5%)	32 (6%)	84 (3%)	217 (6%)	47 (4%)
Mexican	1363 (16%)	125 (24%)	533 (18%)	643 (18%)	62 (5%)
SHLNOS	4801 (58%)	270 (52%)	1875 (63%)	2027 (56%)	629 (52%)
OSSHO	205 (2%)	14 (3%)	49 (2%)	117 (3%)	25 (2%)
SSO	255 (3%)	20 (4%)	115 (4%)	99 (3%)	21 (2%)
Age	68.3 ± 11.0	68.5 ± 11.3	69.2 ± 11.0	67.5 ± 11.0	68.3 ± 11.0
Sex					
Male	86 863 (51%)	6062 (51%)	32 541 (51%)	38 468 (51%)	9792 (51%)
Female	83 464 (49%)	5926 (49%)	31 579 (49%)	36 373 (49%)	9586 (49%)
Income^a^					
<38 000	30 481 (18%)	2211 (18%)	11 174 (17%)	13 441 (18%)	3655 (19%)
38 000‐47 999	40 266 (24%)	3674 (31%)	15 703 (24%)	16 188 (22%)	4701 (24%)
48 000‐62 999	45 359 (27%)	3193 (27%)	17 823 (28%)	18 725 (25%)	5618 (29%)
>63 000	54 221 (32%)	2910 (24%)	19 420 (30%)	26 487 (35%)	5404 (28%)
Education^b^					
>21.0%	28 421 (17%)	2262 (19%)	10 400 (16%)	12 634 (17%)	3125 (16%)
13.0%‐20.9%	44 007 (26%)	3506 (29%)	16 469 (26%)	18 768 (25%)	5264 (27%)
7.0%‐12.9%	56 176 (33%)	4169 (35%)	21 542 (34%)	23 796 (32%)	6669 (34%)
<7%	41 723 (24%)	2051 (17%)	15 709 (24%)	19 643 (26%)	4320 (22%)
Insurance					
Uninsured	5208 (3%)	376 (3%)	1814 (3%)	2460 (3%)	558 (3%)
Private	56 514 (33%)	3521 (29%)	20 125 (31%)	26 690 (36%)	6178 (32%)
Medicaid	8772 (5%)	708 (6%)	2679 (4%)	4269 (6%)	1116 (6%)
Medicare	99 833 (59%)	7385 (62%)	39 502 (62%)	41 422 (55%)	11 526 (59%)
Facility location					
New England	9756 (6%)	994 (8%)	3139 (5%)	5521 (7%)	102 (1%)
Middle Atlantic	28 106 (17%)	1510 (13%)	6870 (11%)	18 004 (24%)	1722 (9%)
South Atlantic	37 966 (22%)	2180 (18%)	14 476 (23%)	12 756 (17%)	8554 (44%)
East North Central	30 593 (18%)	3000 (25%)	9780 (15%)	13 550 (18%)	4263 (22%)
East South Central	11 733 (7%)	755 (6%)	4995 (8%)	4605 (6%)	1378 (7%)
West North Central	13 709 (8%)	925 (8%)	5768 (9%)	6654 (9%)	362 (2%)
West South Central	13 590 (8%)	1039 (9%)	6031 (9%)	5551 (7%)	969 (5%)
Mountain	6552 (4%)	302 (3%)	3602 (6%)	1698 (2%)	950 (5%)
Pacific	18 322 (11%)	1283 (11%)	9459 (15%)	6502 (9%)	1078 (6%)
Distance	40.5 ± 137.1	29.5 ± 137.0	28.5 ± 112.6	55.7 ± 162.3	27.9 ± 90.5
Urban/rural					
Metro	139 504 (82%)	8222 (69%)	52 075 (81%)	62 366 (83%)	16 841 (87%)
Metro adjacent	17 336 (10%)	2128 (18%)	6654 (10%)	7018 (9%)	1536 (8%)
Non‐metro adjacent	6690 (4%)	1057 (9%)	2694 (4%)	2611 (3%)	328 (2%)
Rural	6797 (4%)	581 (5%)	2697 (4%)	2846 (4%)	673 (3%)
Surgery					
No	129 497 (76%)	10 315 (86%)	52 069 (81%)	52 719 (70%)	14 394 (74%)
Yes	40 830 (24%)	1673 (14%)	12 051 (19%)	22 122 (30%)	4984 (26%)
Stage					
Stage = 1	14 172 (8%)	811 (7%)	5380 (8%)	6348 (8%)	1633 (8%)
Stage = 2	51 822 (30%)	2257 (19%)	16 183 (25%)	27 279 (36%)	6103 (31%)
Stage = 3	21 513 (13%)	1238 (10%)	7563 (12%)	10 308 (14%)	2404 (12%)
Stage = 4	82 820 (49%)	7682 (64%)	34 994 (55%)	30 908 (41%)	9238 (48%)
Charlson‐Deyo score					
Score = 0	112 420 (66%)	7874 (66%)	41 473 (65%)	50 936 (68%)	12 137 (63%)
Score = 1	43 596 (25%)	3064 (26%)	16 912 (26%)	18 225 (24%)	5395 (28%)
Score = 2	10 034 (6%)	729 (6%)	4032 (6%)	3997 (5%)	1276 (7%)
Score = 3	4277 (3%)	321 (3%)	1703 (3%)	1683 (2%)	570 (3%)

^a^ Median household income for each patient's area of residence (zip code), based on 2000 US Census data.

^b^ Percentage of adults in the patient's area of residence (zip code) who did not graduate from high school, based on 2000 US Census data.

### 3.2 Factors associated with survival

Patients of Hispanic ethnicity, female sex, higher community income, higher community percentage with high school education, private insurance, lower Charlson‐Deyo score, and earlier stage at diagnosis had improved survival, regardless of facility type, in both univariate and multivariate analyses (Tables 2, 3). In multivariate analysis, once adjusted for other socioeconomic factors, geography, stage, surgical resection, and Charlson‐Deyo score, living in a metropolitan area was associated with improved survival only at ARP. Patients treated at CCCP who live in metropolitan adjacent or non‐metropolitan adjacent have improved overall survival, while patients treated at CCP who live in non‐metropolitan adjacent areas had improved overall survival (Table 3).

**TABLE 2:** Univariate analysis of factors associated with survival

Variable	All facilities	CCP	CCCP	ARP	INCP
	HR (95% CI) *P* Value	HR (95% CI) *P* Value	HR (95% CI) *P* Value	HR (95% CI) *P* Value	HR (95% CI) *P* Value
Race					
Non‐Hispanic White					
Non‐Hispanic Black	1.05 (1.03‐1.06) <0.001	0.99 (0.93‐1.05) 0.781	1.04 (1.01‐1.07) 0.005	1.13 (1.10‐1.15) <0.001	0.99 (0.95‐1.03) 0.479
Hispanic	0.92 (0.90‐0.94) <0.001	0.84 (0.76‐0.92) <0.001	0.98 (0.94‐1.02) 0.291	0.93 (0.89‐0.96) <0.001	0.85 (0.79‐0.90) <0.001
Age	1.02 (1.02‐1.02) <0.001	1.02 (1.02‐1.02) <0.001	1.02 (1.02‐1.02) <0.001	1.02 (1.02‐1.02) <0.001	1.02 (1.02‐1.02) <0.001
Sex					
Male					
Female	0.97 (0.96‐0.98) <0.001	0.95 (0.92‐0.99) 0.010	0.97 (0.96‐0.99) 0.002	0.97 (0.96‐0.99) <0.001	0.93 (0.91‐0.96) <0.001
Income^a^					
<38 000					
38 000‐47 999	0.95 (0.93‐0.96) <0.001	0.95 (0.90‐1.00) 0.059	0.94 (0.92‐0.97) <0.001	0.91 (0.89‐0.93) <0.001	0.98 (0.94‐1.03) 0.448
48 000‐62 999	0.90 (0.89‐0.92) <0.001	0.92 (0.87‐0.97) 0.004	0.91 (0.89‐0.93) <0.001	0.86 (0.84‐0.88) <0.001	0.97 (0.93‐1.02) 0.290
>63 000	0.82 (0.81‐0.83) <0.001	0.88 (0.83‐0.93) <0.001	0.86 (0.84‐0.88) <0.001	0.79 (0.77‐0.81) <0.001	0.92 (0.88‐0.97) <0.001
Education^b^					
>21.0%					
13.0%‐20.9%	0.98 (0.96‐0.99) 0.002	1.01 (0.96‐1.07) 0.704	0.98 (0.96‐1.01) 0.138	0.96 (0.93‐0.98) <0.001	0.99 (0.94‐1.03) 0.551
7.0%‐12.9%	0.93 (0.91‐0.94) <0.001	0.97 (0.92‐1.03) 0.287	0.94 (0.91‐0.96) <0.001	0.89 (0.87‐0.91) <0.001	1.00 (0.95‐1.04) 0.854
<7%	0.86 (0.85‐0.88) <0.001	0.95 (0.89‐1.01) 0.078	0.88 (0.86‐0.90) <0.001	0.84 (0.82‐0.86) <0.001	0.93 (0.89‐0.98) 0.007
Insurance					
Uninsured					
Private	0.77 (0.75‐0.80) <0.001	0.75 (0.67‐0.84) <0.001	0.77 (0.73‐0.81) <0.001	0.78 (0.75‐0.82) <0.001	0.76 (0.69‐0.83) <0.001
Medicaid	0.96 (0.93‐1.00) 0.031	0.91 (0.80‐1.04) 0.178	0.95 (0.89‐1.01) 0.081	1.00 (0.95‐1.06) 0.944	0.91 (0.82‐1.02) 0.090
Medicare	1.04 (1.01‐1.07) 0.018	1.05 (0.94‐1.17) 0.383	1.02 (0.97‐1.08) 0.357	1.01 (0.97‐1.06) 0.647	1.04 (0.95‐1.14) 0.388
Facility location					
New England					
Middle Atlantic	0.87 (0.85‐0.89) <0.001	0.93 (0.86‐1.01) 0.101	1.01 (0.97‐1.06) 0.514	0.86 (0.83‐0.89) <0.001	0.55 (0.45‐0.67) <0.001
South Atlantic	1.02 (0.99‐1.04) 0.190	1.06 (0.98‐1.15) 0.122	1.04 (0.99‐1.08) 0.087	0.97 (0.94‐1.00) 0.072	0.56 (0.46‐0.69) <0.001
East North Central	1.02 (1.00‐1.05) 0.098	1.01 (0.94‐1.09) 0.834	1.00 (0.96‐1.04) 0.903	1.00 (0.97‐1.04) 0.925	0.63 (0.52‐0.77) <0.001
East South Central	1.07 (1.04‐1.10) <0.001	1.08 (0.98‐1.19) 0.114	1.06 (1.01‐1.11) 0.010	1.04 (1.00‐1.09) 0.040	0.60 (0.49‐0.73) <0.001
West North Central	1.00 (0.97‐1.03) 0.904	0.97 (0.88‐1.06) 0.465	1.02 (0.98‐1.07) 0.386	0.96 (0.92‐0.99) 0.018	0.77 (0.62‐0.97) 0.024
West South Central	0.95 (0.92‐0.97) <0.001	0.81 (0.74‐0.89) <0.001	0.95 (0.91‐0.99) 0.020	0.90 (0.87‐0.94) <0.001	0.60 (0.48‐0.73) <0.001
Mountain	0.98 (0.95‐1.02) 0.321	0.86 (0.75‐0.98) 0.024	0.93 (0.88‐0.97) 0.002	0.89 (0.84‐0.94) <0.001	0.64 (0.52‐0.79) <0.001
Pacific	0.99 (0.97‐1.02) 0.684	0.97 (0.89‐1.06) 0.462	0.95 (0.91‐0.99) 0.013	0.94 (0.91‐0.98) 0.002	0.68 (0.55‐0.83) <0.001
Distance	1.00 (1.00‐1.00) <0.001	1.00 (1.00‐1.00) <0.001	1.00 (1.00‐1.00) <0.001	1.00 (1.00‐1.00) <0.001	1.00 (1.00‐1.00) <0.001
Urban/rural					
Metro					
Metro adjacent	1.04 (1.02‐1.06) <0.001	0.96 (0.91‐1.00) 0.072	0.98 (0.96‐1.01) 0.184	1.06 (1.03‐1.09) <0.001	1.03 (0.98‐1.09) 0.204
Non‐metro adjacent	1.05 (1.02‐1.08) <0.001	0.93 (0.87‐0.99) 0.030	0.97 (0.93‐1.01) 0.105	1.08 (1.03‐1.12) <0.001	1.06 (0.94‐1.19) 0.329
Rural	1.02 (1.00‐1.05) 0.068	0.95 (0.87‐1.04) 0.272	1.02 (0.98‐1.07) 0.240	1.03 (0.99‐1.07) 0.187	0.93 (0.86‐1.01) 0.074
Surgery					
No					
Yes	0.32 (0.32‐0.33) <0.001	0.33 (0.31‐0.35) <0.001	0.33 (0.32‐0.33) <0.001	0.33 (0.32‐0.34) <0.001	0.32 (0.31‐0.33) <0.001
Stage					
Stage = 1					
Stage = 2	1.00 (0.98‐1.02) 0.760	0.85 (0.78‐0.93) <0.001	0.98 (0.95‐1.01) 0.223	1.09 (1.06‐1.12) <0.001	0.98 (0.92‐1.04) 0.438
Stage = 3	1.55 (1.51‐1.59) <0.001	1.18 (1.07‐1.30) <0.001	1.43 (1.37‐1.48) <0.001	1.74 (1.68‐1.80) <0.001	1.53 (1.43‐1.64) <0.001
Stage = 4	2.81 (2.76‐2.87) <0.001	2.40 (2.21‐2.60) <0.001	2.65 (2.57‐2.74) <0.001	2.92 (2.83‐3.01) <0.001	2.84 (2.68‐3.01) <0.001
Charlson‐Deyo score					
Score = 0					
Score = 1	1.11 (1.10‐1.13) <0.001	1.17 (1.12‐1.22) <0.001	1.14 (1.12‐1.17) <0.001	1.06 (1.04‐1.08) <0.001	1.13 (1.09, 1.17) <0.001
Score = 2	1.30 (1.28‐1.33) <0.001	1.44 (1.33‐1.55) <0.001	1.33 (1.29‐1.38) <0.001	1.22 (1.18‐1.27) <0.001	1.32 (1.24, 1.40) <0.001
Score = 3	1.66 (1.61‐1.72) <0.001	1.75 (1.56‐1.97) <0.001	1.69 (1.61‐1.78) <0.001	1.58 (1.50‐1.66) <0.001	1.69 (1.55, 1.84) <0.001

^a^ Median household income for each patient's area of residence (zip code), based on 2000 US Census data.

^b^ Percentage of adults in the patient's area of residence (zip code) who did not graduate from high school, based on 2000 US Census data.

**TABLE 3:** Multivariate analysis of factors associated with survival

Variable	All Facilities	CCP	CCCP	ARP	INCP
	HR (95% CI) *P* value	HR (95% CI) *P* value	HR (95% CI) *P* value	HR (95% CI) *P* value	HR (95% CI) *P* value
Race					
Non‐Hispanic White					
Non‐Hispanic Black	0.95 (0.93‐0.96) <0.001	0.95 (0.89‐1.01) 0.098	0.96 (0.93‐0.98) 0.002	0.96 (0.94‐0.98) 0.001	0.97 (0.93‐1.01) 0.180
Hispanic	0.84 (0.82‐0.86) <0.001	0.85 (0.77‐0.94) 0.002	0.96 (0.92‐1.00) 0.036	0.80 (0.77‐0.84) <0.001	0.78 (0.73‐0.84) <0.001
Dominican	0.60 (0.51‐0.72) <0.001	0.64 (0.32‐1.29) 0.219	0.90 (0.58‐1.40) 0.644	0.57 (0.46‐0.70) <0.001	0.79 (0.47‐1.33) 0.375
SCA	0.68 (0.63‐0.75) <0.001	0.79 (0.55‐1.12) 0.181	0.83 (0.70‐0.98) 0.030	0.61 (0.53‐0.69) <0.001	0.75 (0.62‐0.91) 0.004
Cuban	0.84 (0.76‐0.92) <0.001	1.94 (0.92‐4.08) 0.081	1.15 (0.96‐1.38) 0.132	0.90 (0.73‐1.11) 0.321	0.71 (0.62‐0.81) <0.001
Puerto Rican	0.86 (0.77‐0.96) 0.008	1.23 (0.86‐1.76) 0.265	1.13 (0.90‐1.43) 0.280	0.79 (0.68‐0.91) 0.001	0.78 (0.57‐1.07) 0.128
Mexican	0.84 (0.80‐0.90) <0.001	0.84 (0.69‐1.02) 0.079	0.96 (0.87‐1.05) 0.347	0.78 (0.72‐0.86) <0.001	1.04 (0.79‐1.36) 0.801
SHLNOS	0.86 (0.84‐0.89) <0.001	0.81 (0.70‐0.93) 0.002	0.93 (0.88‐0.98) 0.004	0.85 (0.81‐0.90) <0.001	0.80 (0.73‐0.88) <0.001
OSSHO	0.90 (0.78‐1.04) 0.166	1.39 (0.79‐2.44) 0.258	1.47 (1.10‐1.98) 0.010	0.80 (0.66‐0.98) 0.032	0.73 (0.45‐1.12) 0.147
SSO	1.09 (0.95‐1.24) 0.206	0.87 (0.54‐1.41) 0.581	1.25 (1.04‐1.52) 0.019	1.03 (0.83‐1.27) 0.808	0.75 (0.45‐1.25) 0.272
Age	1.02 (1.02‐1.02) <0.001	1.02 (1.02‐1.02) <0.001	1.02 (1.02‐1.02) <0.001	1.02 (1.02‐1.02) <0.001	1.02 (1.02‐1.02) <0.001
Sex					
Male					
Female	0.96 (0.95‐0.97) <0.001	0.92 (0.88‐0.95) <0.001	0.96 (0.95‐0.98) <0.001	0.96 (0.95‐0.97) <0.001	0.94 (0.91‐0.97) <0.001
Income^a^					
<38 000					
38 000‐47 999	0.95 (0.94‐0.97) <0.001	0.96 (0.91‐1.03) 0.245	0.96 (0.93‐0.99) 0.003	0.94 (0.91‐0.96) <0.001	0.96 (0.91‐1.01) 0.135
48 000‐62 999	0.93 (0.91‐0.94) <0.001	0.92 (0.86‐0.99) 0.020	0.94 (0.91‐0.96) <0.001	0.91 (0.88‐0.93) <0.001	0.98 (0.92‐1.03) 0.420
>63 000	0.87 (0.85‐0.89) <0.001	0.86 (0.79‐0.93) <0.001	0.90 (0.87‐0.93) <0.001	0.84 (0.82‐0.87) <0.001	0.93 (0.87‐1.00) 0.042
Education^b^					
>21.0%					
13.0%‐20.9%	1.01 (1.00‐1.03) 0.168	1.01 (0.95‐1.07) 0.741	1.01 (0.98‐1.04) 0.467	1.01 (1.00‐1.03) 0.037	0.99 (0.93‐1.04) 0.594
7.0%‐12.9%	1.00 (0.99‐1.02) 0.625	0.98 (0.92‐1.05) 0.607	0.98 (0.95‐1.01) 0.171	1.01 (0.98‐1.04) 0.372	0.97 (0.91‐1.03) 0.269
<7%	0.96 (0.94‐0.99) 0.001	0.97 (0.89‐1.06) 0.499	0.94 (0.90‐0.97) <0.001	0.98 (0.95‐1.02) 0.342	0.91 (0.85‐0.98) 0.011
Insurance					
Uninsured					
Private	0.82 (0.80‐0.85) <0.001	0.81 (0.72‐0.91) <0.001	0.78 (0.74‐0.82) <0.001	0.85 (0.82‐0.89) <0.001	0.77 (0.70‐0.85) <0.001
Medicaid	0.98 (0.95‐1.02) 0.317	1.00 (0.88‐1.14) 0.995	0.94 (0.89‐1.00) 0.067	1.01 (0.96‐1.07) 0.742	0.96 (0.86‐1.07) 0.415
Medicare	0.86 (0.83‐0.88) <0.001	0.88 (0.79‐0.99) 0.038	0.78 (0.74‐0.82) <0.001	0.90 (0.85‐0.94) <0.001	0.85 (0.77‐0.94) <0.001
Facility location					
New England					
Middle Atlantic	0.87 (0.85‐0.89) <0.001	0.99 (0.91‐1.08) 0.868	0.97 (0.93‐1.02) 0.200	0.85 (0.82‐0.88) <0.001	0.74 (0.60‐0.90) 0.003
South Atlantic	1.06 (1.04‐1.09) <0.001	1.03 (0.95‐1.12) 0.435	1.01 (0.97‐1.05) 0.665	1.06 (1.03‐1.10) <0.001	0.83 (0.68‐1.01) 0.068
East North Central	1.05 (1.02‐1.08) <0.001	1.02 (0.95‐1.10) 0.594	0.98 (0.94‐1.02) 0.321	1.07 (1.03‐1.10) <0.001	0.88 (0.72‐1.08) 0.224
East South Central	1.14 (1.10‐1.17) <0.001	1.11 (1.00‐1.24) 0.047	1.07 (1.02‐1.12) 0.004	1.15 (1.10‐1.20) <0.001	0.89 (0.72‐1.09) 0.250
West North Central	1.03 (1.00‐1.06) 0.032	1.04 (0.94‐1.14) 0.475	1.01 (0.97‐1.06) 0.529	1.02 (0.98‐1.06) 0.368	0.88 (0.71‐1.10) 0.274
West South Central	0.99 (0.97‐1.02) 0.619	0.88 (0.80‐0.97) 0.011	0.95 (0.90‐0.99) 0.018	0.99 (0.95‐1.04) 0.734	0.81 (0.66‐1.00) 0.048
Mountain	1.04 (1.01‐1.08) 0.019	0.97 (0.84‐1.11) 0.633	0.96 (0.92‐1.01) 0.144	1.01 (0.95‐1.07) 0.786	0.92 (0.75‐1.14) 0.459
Pacific	1.04 (1.02‐1.07) 0.001	1.07 (0.98‐1.17) 0.109	0.97 (0.93‐1.01) 0.173	1.02 (0.98‐1.06) 0.417	0.91 (0.74‐1.11) 0.344
Distance	1.00 (1.00‐1.00) <0.001	1.00 (1.00‐1.00) 0.014	1.00 (1.00‐1.00) <0.001	1.00 (1.00‐1.00) <0.001	1.00 (1.00‐1.00) 0.439
Urban/rural					
Metro					
Metro adjacent	1.02 (1.00‐1.03) 0.033	0.97 (0.92‐1.02) 0.294	0.97 (0.94‐1.00) 0.023	1.04 (1.01‐1.07) 0.003	1.03 (0.97‐1.09) 0.379
Non‐metro adjacent	1.01 (0.98‐1.04) 0.416	0.92 (0.86‐0.99) 0.030	0.96 (0.92‐0.99) 0.035	1.07 (1.02‐1.11) 0.004	1.10 (0.98‐1.24) 0.109
Rural	1.01 (0.98‐1.03) 0.639	0.91 (0.83‐1.00) 0.055	1.03 (0.99‐1.08) 0.113	1.01 (0.97‐1.05) 0.726	0.94 (0.87‐1.02) 0.161
Surgery					
No					
Yes	0.44 (0.43‐0.45) <0.001	0.49 (0.46‐0.53) <0.001	0.46 (0.45‐0.48) <0.001	0.42 (0.41‐0.43) <0.001	0.43 (0.41‐0.46) <0.001
Stage					
Stage = 1					
Stage = 2	1.27 (1.24‐1.30) <0.001	1.13 (1.03‐1.24) 0.008	1.25 (1.21‐1.29) <0.001	1.32 (1.28‐1.36) <0.001	1.31 (1.23‐1.39) <0.001
Stage = 3	1.26 (1.23‐1.30) <0.001	1.14 (1.04‐1.26) 0.006	1.25 (1.20‐1.30) <0.001	1.30 (1.26‐1.35) <0.001	1.28 (1.20‐1.38) <0.001
Stage = 4	2.28 (2.23‐2.32) <0.001	2.27 (2.09‐2.46) <0.001	2.31 (2.23‐2.38) <0.001	2.19 (2.12‐2.26) <0.001	2.37 (2.23‐2.52) <0.001
Charlson‐Deyo score					
Score = 0					
Score = 1	1.13 (1.12‐1.15) <0.001	1.12 (1.07‐1.17) <0.001	1.14 (1.12‐1.16) <0.001	1.11 (1.09‐1.13) <0.001	1.15 (1.11‐1.19) <0.001
Score = 2	1.30 (1.27‐1.33) <0.001	1.31 (1.21‐1.42) <0.001	1.31 (1.27‐1.35) <0.001	1.26 (1.22‐1.31) <0.001	1.33 (1.25‐1.41) <0.001
Score = 3	1.64 (1.58‐1.69) <0.001	1.68 (1.50‐1.89) <0.001	1.58 (1.51‐1.67) <0.001	1.61 (1.54‐1.71) <0.001	1.68 (1.54‐1.83) <0.001

^a^ Median household income for each patient's area of residence (zip code), based on 2000 US Census data.

^b^ Percentage of adults in the patient's area of residence (zip code) who did not graduate from high school, based on 2000 US Census data.

### 3.3 Hispanics have the longest median and overall survival

Median survival was longest for Hispanics (7.52 months), compared to NHW (7.29 months) and NHB (6.64 months) (Table 4, Figure S1). Compared to NHW, NHB had worse overall survival (HR = 1.05, 95% CI: 1.03‐1.06*, P* < .001) and Hispanics had better overall survival (HR = 0.92, 95% CI: 0.90‐0.94*, P* < .001), at all facility types (Table 2). After adjusting for socioeconomic variables, geography, stage, surgical resection, and Charlson‐Deyo score, NHB (HR = 0.95, 95% CI: 0.93‐0.96, *P* < .001) had improved overall survival and Hispanics (HR = 0.84, 95% CI: 0.82‐0.86, *P* < .001) had the best comparative outcomes, as compared to NHW (Table 3). In pairwise comparison, the HR of Hispanics compared to NHW was 0.84 (95% CI: 0.82‐0.86, *P* < .001) and compared to NHB was 0.89 (95% CI: 0.87‐0.92, *P* < .001), confirming the findings above. The HR of NHB compared to NHW was 0.95 (95% CI: 0.93‐0.96, *P* < .001) (Table 5).

**TABLE 4:** Median survival in months with corresponding 95% confidence intervals

	All facilities	CCP	CCCP	ARP	INCP
All races	7.23 (7.16‐7.26)	4.93 (4.80‐5.09)	5.85 (5.78‐5.95)	9.07 (8.97‐9.17)	6.90 (6.74‐7.06)
NHW	7.29 (7.23‐7.36)	4.93 (4.80‐5.13)	5.91 (5.85‐6.01)	9.33 (9.20‐9.43)	6.83 (6.67‐7.00)
NHB	6.64 (6.47‐6.77)	4.57 (4.21‐5.06)	5.52 (5.29‐5.75)	7.69 (7.49‐7.92)	6.74 (6.31‐7.20)
Hispanic	7.52 (7.20‐7.79)	6.14 (4.93‐7.16)	5.59 (5.29‐5.98)	9.07 (8.54‐9.59)	8.38 (7.69‐9.20)
Dominican	10.25 (8.02‐13.37)	15.84 (5.13‐NA)	6.01 (3.58‐14.09)	12.55 (8.44‐16.76)	6.11 (3.68‐13.86)
SCA	9.82 (8.11‐11.10)	6.74 (3.25‐13.93)	6.60 (4.83‐9.30)	11.56 (10.05‐13.34)	10.48 (7.10‐14.69)
Cuban	8.71 (7.29‐9.86)	4.44 (1.18‐NA)	5.52 (4.47‐6.44)	7.43 (5.88‐11.01)	11.43 (9.33‐13.40)
Puerto Rican	8.11 (6.80‐9.69)	4.30 (1.64‐6.80)	5.32 (4.37‐7.69)	10.18 (8.94‐12.55)	8.11 (4.07‐13.90)
Mexican	7.26 (6.60‐7.98)	5.26 (4.21‐7.85)	5.55 (5.06‐6.57)	9.17 (8.28‐10.45)	5.42 (4.07‐9.30)
SHLNOS	7.23 (6.77‐7.66)	6.57 (5.29‐8.80)	5.72 (5.22‐6.14)	8.54 (8.18‐9.26)	7.69 (6.31‐8.44)
OSSHO	7.06 (5.52‐8.54)	4.24 (1.87‐NA)	4.34 (2.17‐7.39)	8.74 (6.74‐12.12)	11.10 (3.48‐24.71)
SSO	6.21 (5.19‐8.28)	7.69 (3.75‐24.64)	4.83 (3.84‐7.52)	8.11 (5.65‐10.84)	6.70 (3.52‐23.36)

OSSHO: Other specified Spanish/Hispanic origin; SCA: South or Central American except Brazil; SHLNOS: Spanish NOS, Hispanic NOS and Latino NOS; SSO: Spanish surname only. NA means we cannot obtain the related estimates. Longer follow‐up time or larger sample size is required to estimate the upper limit of the confidence intervals.

**TABLE 5:** Pairwise comparisons between race/ethnic groups

	Estimate	Hazard ratio	95% CI	*P* value
H‐NHW	−0.17	0.84	(0.82‐0.86)	<0.001
NHB‐NHW	−0.06	0.95	(0.93‐0.96)	<0.001
H‐NHB	−0.12	0.89	(0.87‐0.92)	<0.001

To determine survival benefits in particular Hispanic subgroups, we performed similar analyses by self‐reported country or region of origin. Dominicans and South or Central Americans had the best overall survival. The median survival of Dominicans (n = 168) was 10.25 (8.02‐13.37) months (HR = 0.60, 95% CI: 0.51‐0.72, *P* < .001), while median survival of South or Central Americans (n = 667) was 9.82 (8.11‐11.10) months (HR = 0.68, 95% CI: 0.63‐0.75, *P* < .001), at all facilities combined. A large portion of this survival advantage among Dominicans and South or Central American appears attributable to care received at ARP (Tables 3, 4), as they experienced an approximate 40% reduction in risk of death from PDAC, compared to NHW, if care was received at ARP.

### 3.4 Survival benefit of Academic Research Programs

In univariate analysis, Hispanics had the greatest survival benefit at CCP (HR = 0.84, 95% CI: 0.76‐0.92, *P* < .001), followed by INCP (HR = 0.85, 95% CI: 0.79‐0.90, *P* < .001) and ARP (HR = 0.93, 95% CI: 0.89‐0.96, *P* < .001). There was no survival benefit if care was received at CCCP (Table 2). However, when adjusting for socioeconomic factors, geography, stage, surgical resection, and Charlson‐Deyo score, the survival benefit was most pronounced at ARP (HR = 0.80, 95% CI: 0.77‐0.84, *P* < .001) and INCP (HR = 0.78, 95%CI: 0.73‐0.84, *P* < .001). Survival benefit for Hispanics at CCP was unchanged between univariate and multivariate analyses. Hispanics had a statistically significant survival benefit over NHW at CCCP in the multivariate model (HR = 0.96, 95% CI: 0.92‐1.00, *P* = .036). In summary, Hispanics had the greatest survival benefit when they receive care at ARP and INCP (Table 3).

When all programs are combined, NHB have a survival disadvantage compared to NHW (HR = 1.05, 95% CI: 1.03‐1.06, *P* < .001) (Table 2), but once socioeconomic factors, geography, stage, surgical resection, and Charlson‐Deyo score are adjusted for, NHB have a survival benefit over NHW (HR = 0.95, 95% CI: 0.93‐0.96, *P* < .001) (Table 3). This effect is dependent upon the facility type in which care is received. In univariate analysis, NHB and NHW have similar HRs at CCP and INCP. The survival disadvantage among NHB is driven by treatment at CCCP (HR = 1.04, 95% CI: 1.01‐1.07, *P* = .005) and ARP (HR = 1.13, 95% CI: 1.10‐1.15, *P* < .001). In multivariate analysis, the survival benefit of NHB compared to NHW showed that facility type again influences the overall results. There was no significant difference in HR between NHB and NHW at CCP and INCP, while there was a small survival benefit among NHB compared to NHW at CCCP (HR = 0.96, 95% CI: 0.93‐0.98, *P* = .002) and ARP (HR = 0.96, 95% CI: 0.94‐0.98, *P* = .001). NHW and NHB had a survival benefit when care was received at higher volume centers (CCCP and ARP) compared to lower volume centers (CCP) (Table 6). This benefit was most pronounced among NHB who underwent surgery at higher volume centers (HR = 0.75, 95% CI: 0.63‐0.90, *P* = .002) (Table 7). These results show that NHB have a statistically significant survival benefit over NHW when care is received at CCCP and ARP.

**TABLE 6:** The hazard ratios of high volume (CCCP and ARP) vs low volume (CCP) centers (reference group)

	Hazard ratio	95% CI	*P* value
All patients	0.90	0.88‐0.92	<0.001
NHW	0.90	0.88‐0.92	<0.001
NHB	0.91	0.85‐0.96	0.002
Hispanic	0.94	0.85‐1.04	0.215

INCP was excluded from the analysis as this facility type designation includes a combination of both low‐ and high‐volume centers. The reference group for each row includes that race/ethnic group treated at CCP. For example, NHW treated at CCCP and ARP combined were compared to NHW treated at CCP (reference group).

**TABLE 7:** The hazard ratios of high‐volume (CCCP and ARP) vs low‐volume (CCP) centers for patients who underwent surgery

	Hazard ratio	95% CI	*P* value
All patients with surgery	0.93	0.88‐0.99	0.013
NHW patients with surgery	0.96	0.90‐1.01	0.138
NHB patients with surgery	0.75	0.63‐0.90	0.002
Hispanic patients with surgery	0.90	0.69‐1.17	0.428

INCP was excluded from the analysis as this facility type designation includes a combination of both low‐ and high‐volume centers. The reference group for each row includes that race/ethnic group treated at CCP. For example, NHW treated at CCCP and ARP combined were compared to NHW treated at CCP (reference group).

Pairwise comparison with all races combined shows that overall survival was best at ARP compared to all other facilities. INCP also had improved survival compared to community programs (Table 8).

**TABLE 8:** Pairwise comparisons between facility types

	Estimate	Hazard ratio	95% CI	*P* value
ARP‐CCP	−0.17	0.84	(0.82‐0.86)	<0.001
INCP‐CCP	−0.05	0.95	(0.93‐0.97)	<0.001
CCCP‐CCP	−0.03	0.97	(0.95‐0.99)	0.008
ARP‐CCCP	−0.15	0.86	(0.85‐0.87)	<0.001
INCP‐CCCP	−0.02	0.98	(0.96‐0.99)	0.008
ARP‐INCP	−0.12	0.88	(0.87‐0.90)	<0.001

When median survival time across all facility types was analyzed, median survival was 7.52 (7.2‐7.79) months for Hispanics, 7.29 (7.23‐7.36) months for NHW, and 6.64 (6.47‐6.77) months for NHB. All race or ethnic groups had median survival benefits at ARP (Hispanics = 9.07 months, NHW = 9.33 months, NHB = 7.69 months), whereas median survival was shortest at CCP (Hispanics = 6.14 months, NHW = 4.93 months, NHB = 4.57 months). Median survival in Hispanics was also improved at INCP (8.38 months), but non‐Hispanics did not experience a similar benefit if care was received at INCP (Table 4). In summary, survival was improved at ARP based on Cox proportional‐hazard model, pairwise comparison, and median survival time (Figure S2).

### 3.5 Impact of income, education, and surgical resection on survival

Higher median household income (HR = 0.82, *P* < .001), high school education (HR = 0.86, *P* < .001), and surgical resection (HR = 0.32, *P* < .001) improve overall survival at all facility types (Table 2). After adjusting for socioeconomic factors, geography, stage, and Charlson‐Deyo score, income (HR = 0.87, 95% CI: 0.85‐0.89, *P* < .001), education (HR = 0.96, 95% CI: 0.94‐0.99, *P* = .001), and surgical resection (HR = 0.44, 95% CI: 0.43‐0.45, *P* < .001) remain positive contributors to overall survival (Table 3). The positive effect of these covariates was similar across all facility types. These covariates have varying levels of influence on overall survival among the different race/ethnic groups. The improved survival of NHB over NHW was influenced by these factors at all facilities. When these covariates were not included in the model, the HR for NHB at all facilities was 1.02 (95% CI: 1.01‐1.04, *P* = .005) (Table 9), but when included in the model, the HR for NHB was 0.95 (95% CI: 0.93‐0.96, *P* < .001) at all facilities combined (Table 3), suggesting that the survival benefit of NHB was significantly influenced by these variables.

**TABLE 9:** Hazard ratios for race/ethnicity obtained from the multivariate Cox model, after adjusting for all covariates except income level, attainment of high school education, and surgical resection

	NHB vs NHW	Hispanic vs NHW
	HR (95% CI) *P* value	HR (95% CI) *P* value
All facilities	1.02 (1.01‐1.04) 0.005	0.89 (0.87‐0.91) <0.001
CCP	1.00 (0.93‐1.05) 0.699	0.87 (0.79‐0.97) 0.008
CCCP	1.02 (0.99‐1.05) 0.191	1.00 (0.96‐1.04) 0.861
ARP	1.07 (1.04‐1.09) <0.001	0.86 (0.83‐0.89) <0.001
INCP	1.03 (0.98‐1.07) 0.218	0.84 (0.78‐0.90) <0.001

This effect was also influenced by the facility type at which care is received. NHB had a statistically significant survival benefit over NHW at CCCP (HR = 0.96, 95% CI: 0.93‐0.98, *P* = .002) and ARP (HR = 0.96, 95% CI: 0.94‐0.98, *P* = .001) when all covariates are included (Table 3). If income, education, and surgical resection are excluded from the model, NHB no longer had a survival benefit over NHW at ARP (HR = 1.07, 95% CI: 1.04‐1.11, *P* < .009) (Table 9). In summary, higher income, high school education, and surgical resection were protective factors for NHB, but only at ARP.

Higher median household income, high school education, and surgical resection also contribute to the overall survival of Hispanics, but not as profoundly as seen with NHB. When these covariates were included in the model, the HR for Hispanics compared to NHW was 0.84 (95% CI: 0.82‐0.86*; P* < .001) at all facilities (Table 3). Excluding income, education, and surgical resection, the HR was slightly higher at 0.89 (95% CI: 0.87‐0.91, *P* < .001), meaning that these factors had a positive influence on overall survival for Hispanics (Table 9). However, when analyzed by individual facility types, income, education, and surgical resection did not contribute significantly to overall survival of Hispanics compared to NHW.

## 4 DISCUSSION

PDAC remains a devastating disease regardless of patient race and ethnicity; however, overall and median survival are improved at ARP for all races/ethnicities. Hispanics have a survival benefit over NHW, without controlling for covariates, and they have the longest median survival time at all facility types. This aligns with recently published data out of California, which demonstrated that Hispanics were more likely to survive 5 years with unresectable disease than NHW.^16^ When socioeconomic factors, geography, surgical resection, stage, and Charlson‐Deyo score are controlled for, the survival benefit among Hispanics was greater at ARP and INCP compared to community programs. This suggests that Hispanics benefit significantly from seeking care at ARP and INCP. Higher surgical volume, advanced endoscopy, clinical trials, multidisciplinary care, and insurance bundled access schemes may confer an advantage at ARP, but biological variables may contribute. Moaven et al also demonstrated improved survival among Hispanics with resectable pancreatic cancer, but did not stratify their study by facility type or include subgroups of Hispanics.^7^ Hispanic patients are often excluded from analyses of health disparities or grouped together with White patients.^17^, ^18^, ^19^ Given that Hispanic/Latino patients represent approximately 18% of the US population and estimated to grow 24% by 2065,^20^ such a discrepancy in research rigor is unacceptable. In spite of the wide ancestral diversity that underlies Hispanic identity, when they are included in health disparities studies, all subgroups are categorized together. In this study, we report different outcomes across a range of Hispanic subgroups. Dominicans and patients of South or Central American descent had the greatest survival benefit. While Mexicans, Cubans, and Puerto Ricans also experience benefit, the advantage was not as profound. Genomic variants, diet, the microbiota, as well as cultural and psychosocial stress may alter the inflammatory response, PDAC progression, and tumor biology. To our knowledge, this is the first study to focus on PDAC disparities in Hispanic subgroups. While differences are expected, twofold survival advantages in specific patients over NHW patients are unprecedented. This profound difference may be influenced by small sample sizes in particular subgroups. Further understanding survival benefits in Hispanic subgroups with PDAC may lead to survival gains in patients of all ethnic groups through identification of novel genetic variants that may influence screening, prevention, or treatment of PDAC, as well as risk or protective factors that may be modifiable or targetable.

Consistent with national data, NHB patients with PDAC had worse overall survival and shorter median survival at all facilities.^3^, ^4^ When socioeconomic factors, geography, surgical resection, stage, and Charlson‐Deyo score were controlled, NHB had a nearly equivalent survival benefit compared to NHW at higher volume centers (ARP and CCCP). This normalization appears to be driven by median income, education, and surgical resection, rather than by NHB race. Our results regarding the influence of surgical resection among NHB support previously published data.^5^, ^7^, ^8^, ^9^, ^10^, ^21^ This finding highlights the influence of socioeconomic factors on disparities, as well as a need for better understanding of why surgical resection rates are lower among NHB, which could influence the approach health‐care providers take in counseling NHB in their treatment options. Physician‐patient trust is also a key component that may influence resection rates for NHB and the varying Hispanic subgroups.^22^ Just as mixed amounts of physician trust have been reported across the US for various minority and disadvantaged groups, so might different Hispanic subgroups be more trusting and willing to follow physician treatment than others regardless of treatment facility type.

While our study includes national data and is well‐powered, it is not without limitations. Recall and misclassification bias are inherent in most retrospective studies. In addition, patient care and subsequent data collection may be provided by multiple facilities. The INCP facility type is a heterogeneous umbrella network that includes community programs, as well as ARP, making it challenging to draw conclusions about INCP. Income and education are significant factors that affect overall survival of NHB, but these variables in the NCDB are a reflection of a patient's community, based on census data, and may not accurately reflect each patient's socioeconomic status. Additionally, NHB is not further defined ethnically. For example, we are unable to compare the outcomes in patients of African American, Afro‐Caribbean, or African descent. While Hispanic ethnicity is further defined by country or region of origin, the majority of Hispanic patients were “Not Otherwise Specified,” leading to smaller sample sizes and wider confidence intervals for specific groups. Cancer registries reporting to the NCDB depend upon medical records for ascertainment of data, thus race or ethnicity is influenced by patient self‐reporting as well as comprehensiveness of medical records in capturing this information. The NCDB is currently not equipped to reflect multiple ethnic backgrounds in individual patients, so the effect of possible genetic admixing is unknown. There is also a lack of information on whether an individual patient is a recent immigrant, first‐generation immigrant, or whose ancestors have lived in the United States for many generations. A more thorough understanding of race or ethnicity is needed to further elucidate PDAC disparities, including the use of genomic mapping to determine more granular details on ancestral heritage.

In conclusion, we found that Hispanics with PDAC have better overall survival compared to non‐Hispanics at all facilities, but most profoundly at ARP and INCP. More specifically, Dominicans and South or Central Americans have significantly improved survival at ARP. While NHB have the shortest median survival, higher income, high school education, and surgical resection improve their survival at higher volume centers. Further understanding the disproportionate outcomes at various facility types and the roles of income, education, and surgical resection in survival, together with basic science research into the biological mechanisms of cancer disparities, will improve health equity and clinical outcomes for patients with PDAC.

## CONFLICT OF INTEREST

The authors whose names are listed above certify that they have NO affiliations with or involvement in any organization or entity with any financial interest, or nonfinancial interest, in the subject matter or materials discussed in this manuscript. All authors listed above have confirmed agreement with this statement.

## AUTHOR CONTRIBUTIONS

Andrea N. Riner: Conceptualization, data curation, formal analysis, methodology, project administration, writing—original draft, and writing—review and editing. Patrick W. Underwood: Conceptualization, data curation, formal analysis, methodology, writing—review and editing. Kai Yang: Data curation, formal analysis, writing—review and editing. Kelly M. Herremans: Conceptualization, writing—review and editing. Miles E. Cameron: writing—review and editing. Srikar Chamala: Conceptualization, formal analysis, writing—review and editing. Peihua Qiu: Data curation, formal analysis, writing—review and editing. Thomas J. George: Formal analysis, writing—review and editing. Jennifer B. Permuth: Formal analysis, writing—review and editing. Nipun B. Merchant: Formal analysis, writing—review and editing. Jose G. Trevino: Conceptualization, formal analysis, writing—review and editing.

## Supporting information

Supplementary Material

## Data Availability

The data that support the findings of this study are available from the American College of Surgeons and the Commission on Cancer's (CoC) NCDB Participant Use Data File, through an application process to investigators associated with CoC‐accredited cancer programs.

## References

[1] Siegel RL , Miller KD , Jemal A . Cancer statistics, 2019. CA Cancer J Clin. 2019;69(1):7‐34. 30620402 10.3322/caac.21551

[2] Rahib L , Smith BD , Aizenberg R , Rosenzweig AB , Fleshman JM , Matrisian LM . Projecting cancer incidence and deaths to 2030: the unexpected burden of thyroid, liver, and pancreas cancers in the United States. Cancer Res. 2014;74(11):2913‐2921. 24840647 10.1158/0008-5472.CAN-14-0155

[3] NAACCR Fast Stats 2011‐2015 Cancer Incidence Data. https://faststats.naaccr.org; 2019. Accessed May 20, 2019.

[4] National Program of Cancer Registries and Surveillance, Epidemiology, and End Results SEER*Stat Database: NPCR and SEER Incidence – U.S. Cancer Statistics 2005–2015 Public Use Research Database, United States Department of Health and Human Services, Centers for Disease Control and Prevention and National Cancer Institute. Released June 2018, based on the November 2017 submission; www.cdc.gov/cancer/uscs/public‐use. Accessed May 20, 2019.

[5] Nipp R , Tramontano AC , Kong CY , et al. Disparities in cancer outcomes across age, sex, and race/ethnicity among patients with pancreatic cancer. Cancer Med. 02. 2018;7(2):525‐535. 10.1002/cam4.1277 PMC5806100 29322643

[6] Tavakkoli A , Singal AG , Waljee AK , et al. Racial disparities and trends in pancreatic cancer incidence and mortality in the United States. Clin Gastroenterol Hepatol. 2019;18:171‐178. 31202981 10.1016/j.cgh.2019.05.059

[7] Moaven O , Richman JS , Reddy S , Wang T , Heslin MJ , Contreras CM . Healthcare disparities in outcomes of patients with resectable pancreatic cancer. Am J Surg. 2019;217(4):725‐731. 30583797 10.1016/j.amjsurg.2018.12.007

[8] Lutfi W , Zenati MS , Zureikat AH , Zeh HJ , Hogg ME . Health disparities impact expected treatment of pancreatic ductal adenocarcinoma nationally. Ann Surg Oncol. 2018;25(7):1860‐1867. 29691733 10.1245/s10434-018-6487-5

[9] Murphy MM , Simons JP , Hill JS , et al. Pancreatic resection: a key component to reducing racial disparities in pancreatic adenocarcinoma. Cancer. 2009;115(17):3979‐3990. 19514091 10.1002/cncr.24433

[10] Tohme S , Kaltenmeier C , Bou‐Samra P , Varley PR , Tsung A . Race and health disparities in patient refusal of surgery for early‐stage pancreatic cancer: an NCDB Cohort Study. Ann Surg Oncol. 2018;25(12):3427‐3435. 30043318 10.1245/s10434-018-6680-6

[11] Chu QD , Zhou M , Peddi P , et al. Influence of facility type on survival outcomes after pancreatectomy for pancreatic adenocarcinoma. HPB (Oxford). 2017;19(12):1046‐1057. 28967535 10.1016/j.hpb.2017.04.017

[12] Surgeons ACo . National Cancer Database. https://www.facs.org/quality‐programs/cancer/ncdb, 2019.

[13] Charlson ME , Pompei P , Ales KL , MacKenzie CR . A new method of classifying prognostic comorbidity in longitudinal studies: development and validation. J Chronic Dis. 1987;40(5):373‐383. 3558716 10.1016/0021-9681(87)90171-8

[14] Deyo RA , Cherkin DC , Ciol MA . Adapting a clinical comorbidity index for use with ICD‐9‐CM administrative databases. J Clin Epidemiol. 1992;45(6):613‐619. 1607900 10.1016/0895-4356(92)90133-8

[15] Team RC . R: A Language and Environment for Statistical Computing. Vienna, Austria: R Foundation for Statistical Computing; 2017.

[16] Kardosh A , Lichtensztajn DY , Gubens MA , Kunz PL , Fisher GA , Clarke CA . Long‐term survivors of pancreatic cancer: a California population‐based study. Pancreas. 2018;47(8):958‐966. 30074526 10.1097/MPA.0000000000001133 PMC6095724

[17] Zhou H , Zhang Y , Wei X , et al. Racial disparities in pancreatic neuroendocrine tumors survival: a SEER study. Cancer Med. 2017;6(11):2745‐2756. 28980417 10.1002/cam4.1220 PMC5673917

[18] Wright MJ , Overton HN , Teinor JA , et al. Disparities in the use of chemotherapy in patients with resected pancreatic ductal adenocarcinoma. J Gastrointest Surg. 2019 10.1007/s11605-019-04311-z 31270718

[19] Longnecker DS , Karagas MR , Tosteson TD , Mott LA . Racial differences in pancreatic cancer: comparison of survival and histologic types of pancreatic carcinoma in Asians, blacks, and whites in the United States. Pancreas. 2000;21(4):338‐343. 11075987 10.1097/00006676-200011000-00003

[20] Pew Research Center . Modern Immigration Wave Brings 59 Million to U.S., Driving Population Growth and Change Through 2065: Views of Immigration’s Impact on U.S. Society Mixed. Washington, DC.; 2015.

[21] Bilimoria KY , Bentrem DJ , Ko CY , Stewart AK , Winchester DP , Talamonti MS . National failure to operate on early stage pancreatic cancer. Ann Surg. 2007;246(2):173‐180. 17667493 10.1097/SLA.0b013e3180691579 PMC1933550

[22] Armstrong K , Ravenell KL , McMurphy S , Putt M . Racial/ethnic differences in physician distrust in the United States. Am J Public Health. 2007;97(7):1283‐1289. 17538069 10.2105/AJPH.2005.080762 PMC1913079

