# Supplementary material for: Disparities in Pancreatic Ductal Adenocarcinoma—The Significance of Hispanic Ethnicity, Subgroup Analysis, and Treatment Facility on Clinical Outcomes
Source: Cancer Med. 2020 Apr 13;9(12):4069–82. doi: 10.1002/cam4.3042 (PMC7300394; doi:10.1002/cam4.3042)

Supplemental Figure 1: Survival curve for facility types, based on race/ethnicity. A) Kaplan-Meier survival curves generated from the patients treated at CCP (log-rank test: P=0.002). B) Survival curves for patients treated at CCCP (P=0.009). C) Survival curves for patients treated at ARP (P<0.001). D) Survival curves for patients treated at INCP (P<0.001).

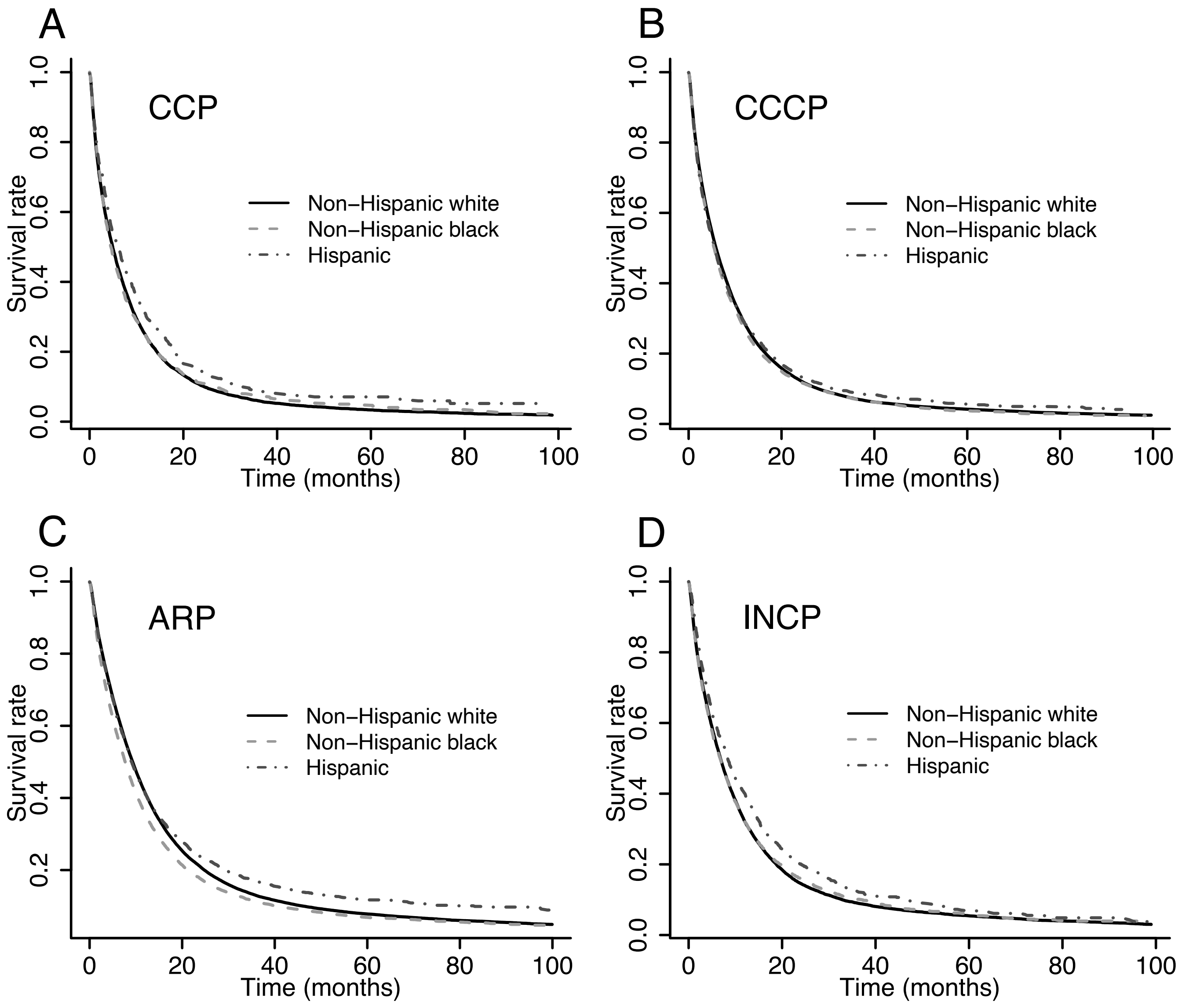

Supplemental Figure 2: Survival curves for race/ethnicity, based on facility type. A) Kaplan-Meier survival plots generated from Non-Hispanic white patients (log-rank test: P<0.001). B) Survival plots for Non-Hispanic black patients (P<0.001). C) Survival plots for Hispanic patients (P<0.001).

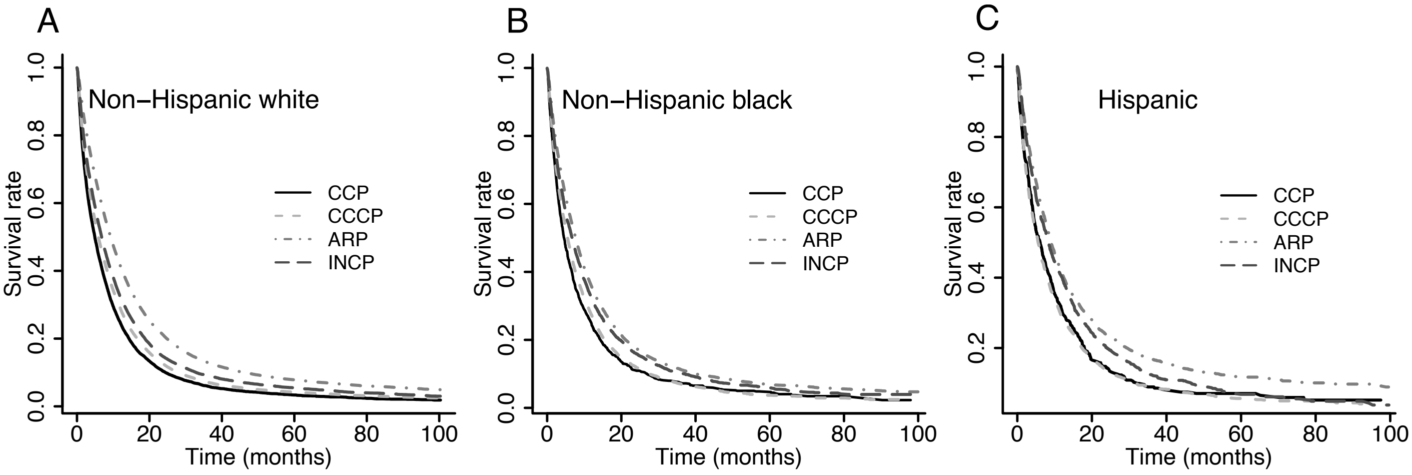

Supplement: Supplementary file 1 — Supplementary Material [file CAM4-9-4069-s001.pdf]
